# Supplementary material for: Characterizing cell-type spatial relationships across length scales in spatially resolved omics data
Source: Nat Commun. 2025 Jan 3;16:350. doi: 10.1038/s41467-024-55700-1 (PMC11699133; doi:10.1038/s41467-024-55700-1)
Supplement: Supplementary file 3 — Supplementary Software 1 [file 41467_2024_55700_MOESM3_ESM.zip › CRAWDAD/plots/supplementary/S7_cluster_corr_heatmap_PKHL_vs_XXCD.pdf]

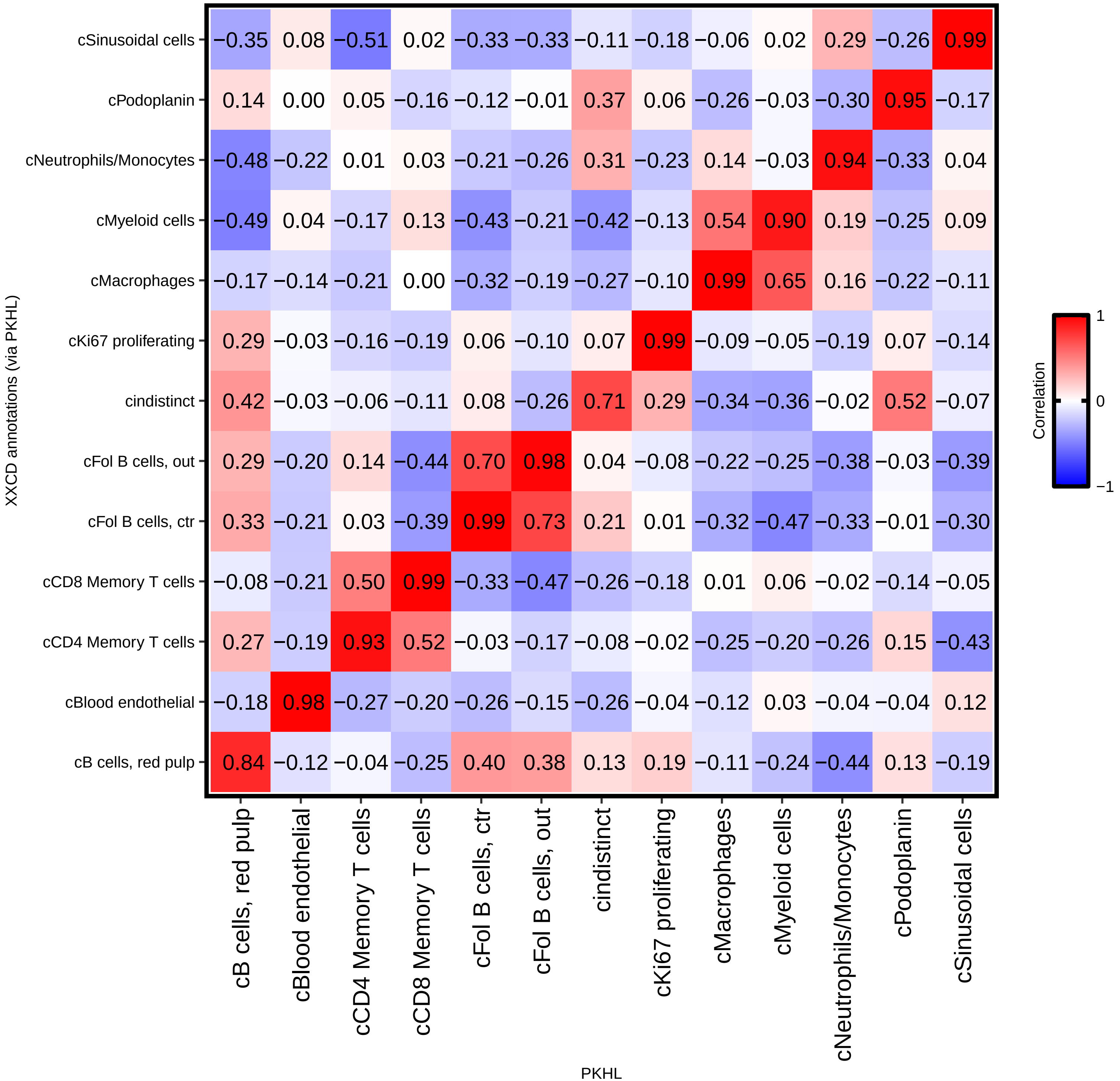
XXCD annotations (via PKHL)
cSinusoidal cells
−0.35 0.08 −0.51 0.02 −0.33 −0.33 −0.11 −0.18 −0.06 0.02 0.29 −0.26 0.99
cPodoplanin
0.14 0.00 0.05 −0.16 −0.12 −0.01 0.37 0.06 −0.26 −0.03 −0.30 0.95 −0.17
cNeutrophils/Monocytes
−0.48 −0.22 0.01 0.03 −0.21 −0.26 0.31 −0.23 0.14 −0.03 0.94 −0.33 0.04
cMyeloid cells
−0.49 0.04 −0.17 0.13 −0.43 −0.21 −0.42 −0.13 0.54 0.90 0.19 −0.25 0.09
cMacrophages
−0.17 −0.14 −0.21 0.00 −0.32 −0.19 −0.27 −0.10 0.99 0.65 0.16 −0.22 −0.11
cKi67 proliferating
0.29 −0.03 −0.16 −0.19 0.06 −0.10 0.07 0.99 −0.09 −0.05 −0.19 0.07 −0.14
cindistinct
0.42 −0.03 −0.06 −0.11 0.08 −0.26 0.71 0.29 −0.34 −0.36 −0.02 0.52 −0.07
cFol B cells, out
0.29 −0.20 0.14 −0.44 0.70 0.98 0.04 −0.08 −0.22 −0.25 −0.38 −0.03 −0.39
cFol B cells, ctr
0.33 −0.21 0.03 −0.39 0.99 0.73 0.21 0.01 −0.32 −0.47 −0.33 −0.01 −0.30
cCD8 Memory T cells
−0.08 −0.21 0.50 0.99 −0.33 −0.47 −0.26 −0.18 0.01 0.06 −0.02 −0.14 −0.05
cCD4 Memory T cells
0.27 −0.19 0.93 0.52 −0.03 −0.17 −0.08 −0.02 −0.25 −0.20 −0.26 0.15 −0.43
cBlood endothelial
−0.18 0.98 −0.27 −0.20 −0.26 −0.15 −0.26 −0.04 −0.12 0.03 −0.04 −0.04 0.12
cB cells, red pulp
0.84 −0.12 −0.04 −0.25 0.40 0.38 0.13 0.19 −0.11 −0.24 −0.44 0.13 −0.19
cB cells, red pulp
cBlood endothelial
cCD4 Memory T cells
cCD8 Memory T cells
cFol B cells, ctr
cFol B cells, out
cindistinct
cKi67 proliferating
cMacrophages
cMyeloid cells
cNeutrophils/Monocytes
cPodoplanin
cSinusoidal cells
PKHL
Correlation
1
0
−1
